# Network Models of Frequency Modulated Sweep Detection

**DOI:** 10.1371/journal.pone.0115196

**Published:** 2014-12-16

**Authors:** Steven Skorheim, Khaleel Razak, Maxim Bazhenov

**Affiliations:** 1 Department of Cell Biology and Neuroscience, University of California Riverside, Riverside, California, United States of America; 2 Psychology Department, University of California Riverside, Riverside, California, United States of America; McGill University, Canada

## Abstract

Frequency modulated (FM) sweeps are common in species-specific vocalizations, including human speech. Auditory neurons selective for the direction and rate of frequency change in FM sweeps are present across species, but the synaptic mechanisms underlying such selectivity are only beginning to be understood. Even less is known about mechanisms of experience-dependent changes in FM sweep selectivity. We present three network models of synaptic mechanisms of FM sweep direction and rate selectivity that explains experimental data: (1) The ‘facilitation’ model contains frequency selective cells operating as coincidence detectors, summing up multiple excitatory inputs with different time delays. (2) The ‘duration tuned’ model depends on interactions between delayed excitation and early inhibition. The strength of delayed excitation determines the preferred duration. Inhibitory rebound can reinforce the delayed excitation. (3) The ‘inhibitory sideband’ model uses frequency selective inputs to a network of excitatory and inhibitory cells. The strength and asymmetry of these connections results in neurons responsive to sweeps in a single direction of sufficient sweep rate. Variations of these properties, can explain the diversity of rate-dependent direction selectivity seen across species. We show that the inhibitory sideband model can be trained using spike timing dependent plasticity (STDP) to develop direction selectivity from a non-selective network. These models provide a means to compare the proposed synaptic and spectrotemporal mechanisms of FM sweep processing and can be utilized to explore cellular mechanisms underlying experience- or training-dependent changes in spectrotemporal processing across animal models. Given the analogy between FM sweeps and visual motion, these models can serve a broader function in studying stimulus movement across sensory epithelia.

## Introduction

A frequency modulated (FM) sweep is an auditory version of a broad class of sensory inputs generated by stimulus motion across the sensory epithelium. FM sweeps are common in animal vocalizations including human speech. FM sweeps are important in speech discrimination [1], [2], [3] and deterioration of FM detection with presbycusis is correlated with speech recognition deficits [4], [5]. As found in the visual and somatosensory systems, auditory system neurons are selective for the rate (speed) and/or direction of such motion. A broad range of FM sweep rate-dependent direction selectivity is found across animal species [6] [7] [8], [9], [10], [11], but the synaptic/network properties that generate this diversity in spectrotemporal processing are unclear. The development of FM sweep selectivity is experience-dependent [12], but the plasticity mechanisms are unknown. To address these issues, we developed network models of three synaptic mechanisms that explain experimental data (reviewed in [13]), and explored plasticity mechanisms responsible for development of direction selectivity.

The first mechanism is asymmetric sideband inhibition [14], [15], [16]. The timing and strength of sideband inhibition relative to excitation shapes FM sweep selectivity [9], [17], [18], [19], [20]. A second mechanism for FM sweep selectivity is facilitation [21], [22]. Individual cells receive sub-threshold excitation from two tones of different frequencies. Direction/rate selectivity emerges because only one sequence of tones generates the appropriate coincidence that is necessary for spike generation. The third mechanism is duration tuning for tones. Duration tuning predicts FM rate selectivity [23], [24]. Coincidence of a rebound from inhibition and a delayed excitation underlie duration tuning in this model [25]. Alternate models that do not depend on a coincidence mechanism have also been proposed [26]. Here we use a network model to evaluate the synaptic properties that cause a dependence on coincidence mechanisms. Different brain regions may utilize each of these mechanisms separately or combine them for efficient spectrotemporal processing. The main goal of this study was to implement and compare these mechanisms in biologically feasible network settings.

These models serve to test theories that explain changes in spectrotemporal processing due to formal training [27] or developmental experience [21]. Although development of FM sweep selectivity is experience-dependent, the underlying synaptic mechanisms of plasticity are not known [21]. STDP mechanisms have been proposed to underlie experience-dependent plasticity of visual motion selectivity in the optic tectum [28]. Repeated presentation of a motion direction caused neurons to develop direction selectivity. This was shown to be dependent on the velocity of movement and STDP. While it has been proposed that STDP shapes the development of FM sweep selectivity [21], it is unclear what network parameters underlie such plasticity. Therefore, the second goal of this study was to determine if and how STDP shapes experience-dependent changes in FM sweep direction selectivity.

## Results

### Sideband inhibition

**Figure 1:**
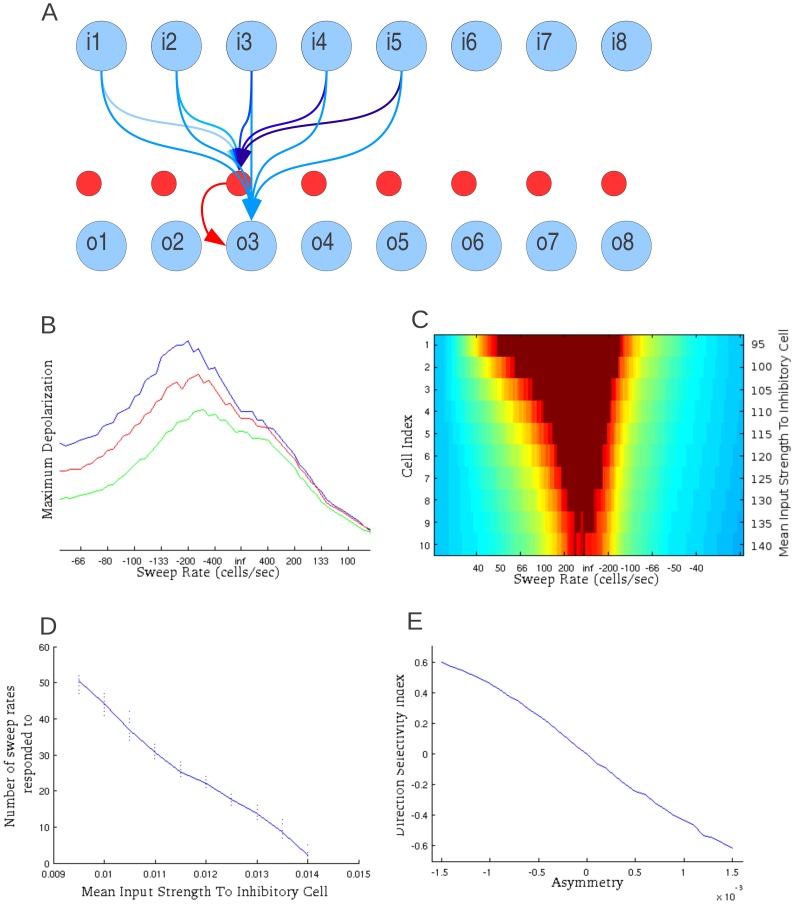
Sideband inhibition mechanism of FM sweep detection. A) Network layout. This model uses asymmetric inhibitory sidebands to differentiate between sweeps of different rates and directions. This network is sensitive to sweeps in preferred direction that are of the target rate or faster resulting in so-called ‘Fast-pass’ rate selective neurons. Excitation is in blue; inhibition is in red. Sweep rate selectivity is dependent upon the strength of synaptic inputs to the inhibitory layer. Direction selectivity depends on the asymmetry of these inputs A sweep of sufficient speed will excite the output cell to fire before the inhibitory interneuron can suppress the output. Although the excitation to the inhibitory cells is generally stronger their route to the output cells is one synapse longer allowing the output cell to receive sufficient excitation before inhibition can prevent cell firing. B) The level of excitation in output cells in response to sweeps of various rates. Spikes are prevented to show depolarization. The green line represents the least sensitive cell (cell 10). As these lines do not cross there is no sweep rate which will evoke a response in the cell represented by the green line that does not also evoke a response in the cells represented by the other two lines. The X-axis shows sweep rate in cells per second in an arbitrarily chosen positive direction. Negative sweep rates represent sweeps in the other direction. ‘Inf’ indicates all input cells fire simultaneously. C) This two dimensional plot shows maximum excitation of 10 output cells in response to a full range of sweep rates. Sweep rates are varied across the x-axis with each output cell arranged along the y-axis. Color indicates maximum excitation during the sweep while spikes are presented in deep red. The asymmetry or the response of the network is directly related to the asymmetry of the strength of connections from the input layer to the inhibitory layer. D) This shows range of sweep rates a given output cell responds to as a function of the mean strength of connections from the input layer to the inhibitory cell. The range is measured by the number of sweep rates that evoke a response out of the total test set. Dots represent individual trials with various Asymmetry coefficients. E) Direction Selectivity Index (DSI) is shown in comparison to the degree of asymmetry of the strengths of connections from the input cells to the inhibitory cell.

Auditory neuron receptive fields contain excitatory and inhibitory components that are approximately spectrally balanced [29], [30]. The layout of the model that captures the spectral balance is shown in Fig. 1A. A number of consecutive frequency sensitive input cells send excitatory input to an inhibitory cell and an output cell. Each input cell responds to a particular frequency; therefore, a sweep leads to sequential activation of the input cells. The inhibitory cell provides feed-forward inhibition to the output cell [31]. Thus, the output cell requires several (arriving close in time) excitatory inputs to spike. Sweeps of sufficiently fast rate lead to summation of excitatory inputs in the output cell making it fire before the inhibitory cell could prevent the response. Although the excitation to the inhibitory cells was generally stronger [32], their route to the output cells was one synapse longer allowing the output cell to receive sufficient excitation before inhibition could prevent cell firing.

In the model, as a sweep activated successive tone-selective cells, these cells begin to excite the inhibitory cell. As there are direct excitatory connections from the input cells to the output cells the excitation arrived faster than the inhibition. For fast sweeps this resulted in an output cell that was well on its way to spiking before inhibition arrived. On the other hand, for sweeps slower than a threshold sweep rate, feed-forward inhibitory input reached the output cell before it received enough excitatory inputs from the input neurons to spike. Therefore, inhibition would prevent spiking.

Any output cell that responded to a given sweep rate would also respond to sweeps faster than that, giving rise to the ‘fast-pass’ type rate selectivity function [33]. In this model, output cells could be made responsive to a narrower range of sweep rates by increasing excitatory input to inhibitory cells (Fig. 1C). Thus, output cells receiving strongest inhibition would respond only to the fastest sweeps in a given set and therefore will be tuned to a narrow range of sweep rates (Fig. 1D). In contrast, the cells that received weakest inhibitory input would be less selective and would respond to a broader range of sweeps (Fig. 1C, Fig. 1D). Thus, by adjusting the excitatory input strength upon inhibitory neurons, a broad range of rate selectivity functions can be generated. Maximum excitation of three output cells is shown in Fig. 1B (to illustrate the temporal profile of the response, the cells were prevented from spiking). The least selective cells always respond more strongly than the more selective ones regardless of sweep rate. The maximum excitation was asymmetrical due to asymmetry in the strength of connections from the input cells to the inhibitory cell.

In general, this model would respond to sweeps of both directions, however, direction preference is introduced by implementing asymmetry of synaptic strengths in the connections from the input cells to the inhibitory cells [9], [20]. The asymmetry was calculated by subtracting the distance weighted average of synaptic strength of connections from the input cells to the inhibitory cells on one side of the central tone-responsive input cell from those on the other side. 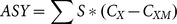
Where ASY is asymmetry, S is synaptic strength, 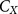 is the position of an input cell that sends a connection to the inhibitory cell and 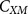 is the position of the input cell in the center of those that send connections to the inhibitory cell.

When this factor was set to zero, all connections from the cells that responded to the low frequency range (the cells on the left in the Fig. 1A) were of the same strength as those from the high frequency range (the cells on the right in the Fig. 1A). Under this condition the network had no direction preference. When the asymmetry was non-zero, the synaptic strength of inputs from the cells responsive to one end of the frequency spectrum was higher than those from the other end. To quantify this, we introduced direction selectivity index (DSI) as a measure of how strongly the network prefers sweeps in one direction over the other. It is defined by the number of sweeps the network responds to inputs in one direction subtracted from the number of sweeps responded to the inputs in the other direction and divided by the total number of sweeps it responded to. 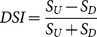
Where 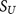 is the number of upward sweeps the network responds to 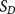 is the number of downward sweeps the network responds to.

Fig. 1E shows that DSI increases with the asymmetry in the network. Thus, manipulation of two properties of this network, asymmetry (Fig. 1E) and strength of input to the inhibitory cells (Fig. 1D), can generate a broad range of rate-dependent direction selectivity functions. Importantly, this also indicates that measures of direction selectivity in experiments must be taken at different sweep rates.

**Figure 2:**
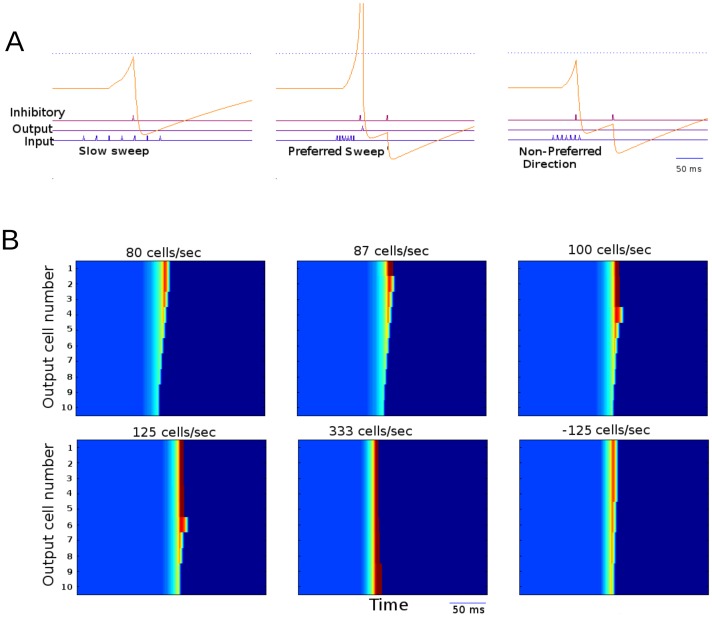
Sideband inhibition network response to a variety of sweeps. A) Voltage traces of an output cell in response to a variety of sweep rates. The cells firing threshold is shown as a dotted blue line. The lower traces indicate spikes of all input cells (bottom) the shown output cell (middle) and the inhibitory interneuron (upper). During sweeps of non-preferred rates the network goes above normal spiking threshold and is only prevented from spiking by inhibition. B). A raster plot of output cell excitation over time for cells with different preferred sweep rates for 6 different sweeps. The cells depolarization is represented by color. Cells are arranged along the y-axis time is represented on the X-axis. Each frame represents the output cells' response to a single sweep with the sweep rate indicated above each panel. The lower numbered cells are responsive to a broader range extending to progressively slower sweeps. In the slowest sweep no cells fire in response to the input (shown by the deepest red). Faster sweeps cause progressively more cells to fire until the sweep at 333 cells/second elicits spikes in all of the output cells. Comparing this to Fig. 5B we can see that this mechanism operates within much shorter timescales than coincidence detection.

Fig. 2 shows the response of the output cell to individual sweeps. It illustrates that for slow sweeps, the inhibitory cells fired before sufficient number of excitatory inputs triggered an output cell response (Fig. 2A, left). For sweeps of sufficient rate the inhibitory cell spiked too late to prevent output spike (Fig. 2A, middle). Direction selectivity in this model was determined by asymmetry in the strength of connections from the input layer to the inhibitory layer. The network was less sensitive to sweeps coming from the direction that contained stronger connections to the inhibitory cells. Therefore, even for fast sweeps in non-preferred direction, the inhibitory cell could fire early enough to prevent spiking in the output neuron (Fig. 2A, right).

To illustrate response of an ensemble of neurons, Fig. 2B presents responses of 10 different cells (each cell could belong, e.g., to a different network like that illustrated in Fig. 1A), each tuned to a different sweep rate range, as a heat chart. The difference in tuning properties was achieved by adjusting the excitatory input strength upon inhibitory neurons. Six sweeps of different rates were tested; the color represents the excitation of the cell at a particular time. Blue indicates the resting state with dark red representing a spike. The sweep of the slowest rate triggered response only in a cell from the circuit with broadest tuning profile. All neurons spiked in response to the fastest sweep rate.

### STDP shapes direction selectivity in the sideband inhibition model

**Figure 3:**
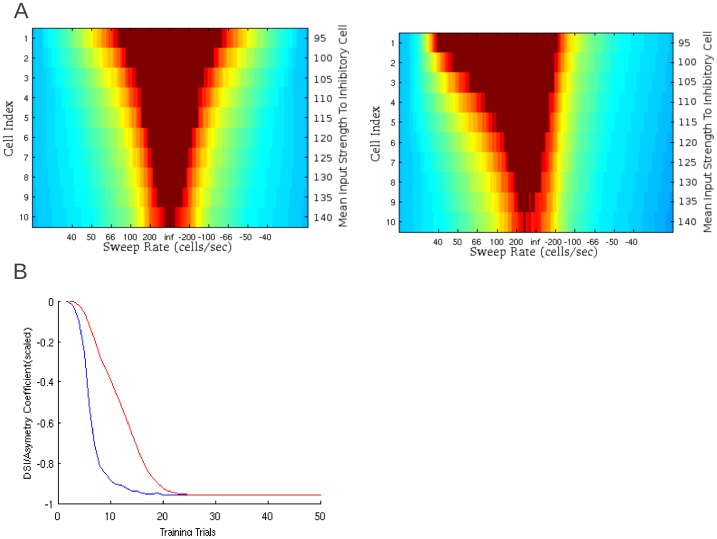
Output response of sideband inhibition model. A) Maximum depolarization state attained in the output cells in response to sweeps of a given rate before and after training with sweeps of a single rate in the preferred direction. Shows the development of asymmetry of response properties due to the effects of STDP learning. B) DSI shown in blue compared to asymmetry index shown in red. As the network is trained by repeated exposure to sweeps of the same rate. Asymmetry index is arbitrarily scaled.

In order to explore how this system might develop direction selectivity *in vivo*, STDP rules were instituted in a series of initially symmetrical networks. STDP was introduced to the excitatory connection from the input layer to the inhibitory layer. This STDP mechanism included the balancing of incoming connections for all cells so that total incoming synaptic strength remained constant. When the network was presented with repeated sweeps in a single direction the networks became selective for sweeps in that direction (Fig. 3). As shown in Fig. 3A over the course of training the profile of responses changes for all cells though the least selective cells are affected the most. It was found that over the course of repeated exposure to sweeps of a given direction, the coefficient of asymmetry increased (by absolute value) along with the DSI (Fig. 3B). Small changes in synaptic asymmetry were able to cause large changes in DSI. Asymmetry continued to increase until the inhibitory cell began to spike before the last input cell at which point the order of STDP was reversed for that cell and asymmetry was reduced. DSI, experiencing a ceiling effect, was not affected by the reduction in synaptic asymmetry.

### Facilitation model

This model is based on the finding that a neuron's spiking response to individual tones is poor or absent, but FM sweep response is robust [21]. When two tones are presented in an appropriately delayed sequence, the spiking response is significantly stronger than the sum of response to individual tones [21], [22], [34]. Direction selectivity for upward sweeps will emerge if an ascending, but not descending, tone sequence produces facilitation. Rate-dependent response will emerge if only specific delays between tones produce facilitation (see Methods).

**Figure 4:**
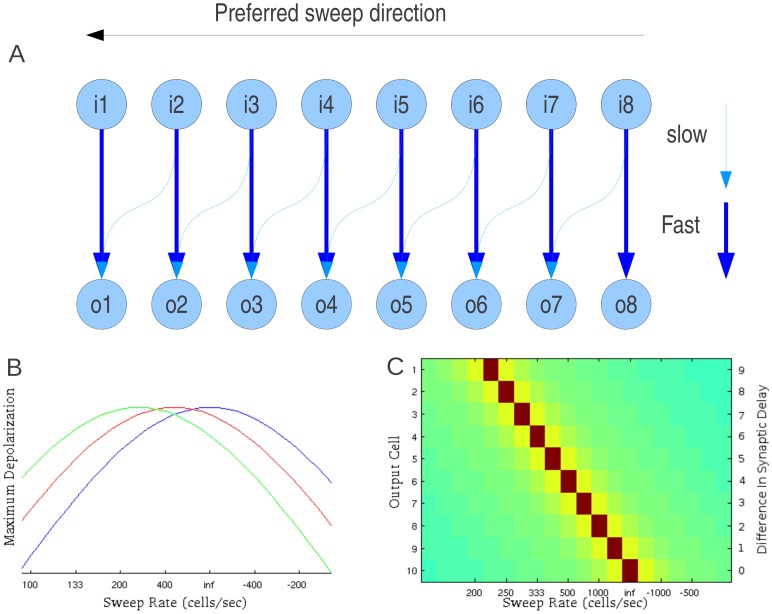
Coincidence-based mechanism of FM sweep detection. This mechanism is also described as the ‘facilitation’ mechanism in the literature [21], [68]. A) Network layout. The input layer (‘i’) represents an array of frequency sensitive cells in a tonotopic organization. The output cells (‘o’) will respond only if the delay between the activation the input cells is of the correct duration to compensate for their different synaptic delays. Sweep direction is shown by the black arrow. The thick blue arrows represent synaptic connections with short delays while the thin blue arrows represent synapses with longer delays. B) The maximum level of excitation achieved by output cells during a sweep as a function of delay between inputs being received by any two neighboring cells (this is inversely proportional to sweep rate). Networks tuned to different best sweep rates are shown in different colors. Here, spikes have been prevented to show excitation. Rates are shown in terms of the number of tonotopic cells stimulated in a second in a given direction. If all cells are stimulated simultaneously the rate is infinite. A negative rate indicates the cells being stimulated in the opposite direction. C) This two dimensional plot shows maximum excitation of 10 output cells in response to a full range of sweep rates. Sweep rates are varied along the x-axis with each output cell arranged along the y-axis. Color indicates maximum excitation during the sweep with spikes represented in deep red.

The network structure is shown schematically in Fig. 4A. As with the sideband model, it contains an input layer with a series of frequency selective cells. Each cell in the output layer received input from cells sensitive to two different frequencies. The synaptic connections from the input cells had different delays. The output cells responded only if the delay between the spike times of two input cells was within a summation sensitive window to compensate for the difference in synaptic delays. As a sweep moved from one frequency to another it activated one tone-selective input cell at a time. When the sweep had a “correct” rate and direction, the slow synapse was activated before the fast synapse and the difference in spike times was the same as the difference between the two delays. It led to summation of inputs and triggered response in the output cells. For simplicity, fast and slow connections have been implemented to be of equal strength though this is not necessary for the functionality of the detector.

This model can be tuned to the sweeps of different rate by changing difference in synaptic delay between neighboring cells. Fig. 4B shows three output cells from different networks (with different combination of delays) each tuned to particular sweep rate. To estimate the maximum excitation achieved by the output cells in response to sweeps of various rates, spike generation was blocked in the output cells. The maximum excitation is a smooth symmetrical curve that peaks at the cells' preferred sweep rate. Fig. 4C shows output cell response (heat chart) as a function of the sweep rate (X-axis) and synaptic delays (Y-axis). In this figure, spiking is not prevented and as such ‘red’ indicates spiking. Each sweep rate triggered response in the output cell only for one specific combination of delays. Furthermore, in contrast to sideband inhibition model, sweeps that were faster than preferred rate did not trigger a response, and this model generates ‘band-pass’ type rate selective functions [33].

**Figure 5:**
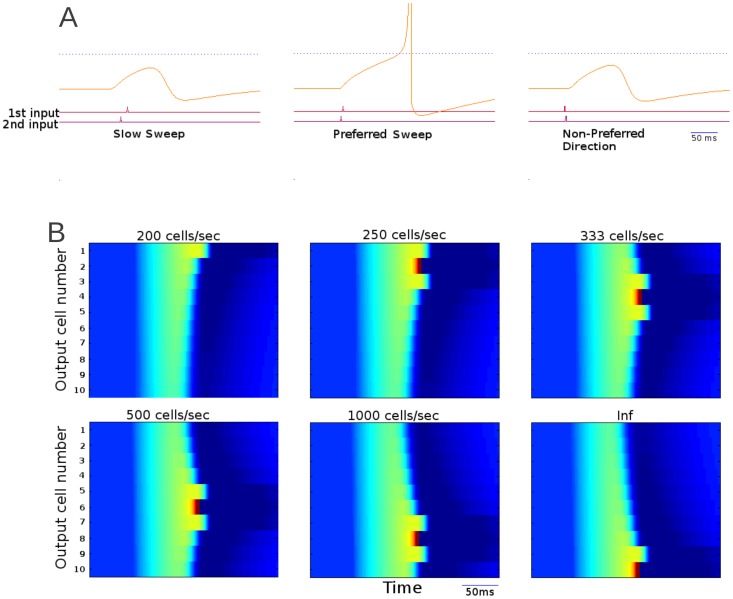
Coincidence detection network response to a variety of sweeps. A) Voltage traces of an output cell in response to a variety of sweep rates. The cells firing threshold is shown in blue. The red and purple traces below show the timing of spikes in the two presynaptic input cells. B) A raster plot of output cell excitation over time for cells with different preferred sweep rates. The cells’ depolarization is represented by color. Output cells are arranged along the y-axis by preferred sweep rate. Time is represented along the y-axis. Each frame represents the response to a single sweep with a specific rate. The higher numbered cells are responsive to faster sweeps. ‘INF’ indicates simultaneous activation of all input cells.

Fig. 5 shows the response of different coincidence detecting cells to individual sweeps as a function of time. In Fig. 5A the first sweep (left panel) was too slow to trigger response. The next sweep (middle panel) was of the preferred rate and triggered spiking. Finally, the last sweep (right panel) had a wrong direction and produced no response. Fig. 5B shows the responses of 10 cells from different networks (each tuned to different preferred rate using different synaptic delays) as a heat chart. In this figure it can be seen that each cell responds to a sweep of its preferred rate in a very similar way that other cells respond to sweeps of their own respective preferred rate. This model presents an intuitive and reliable relationship between synaptic delay and preferred sweep rate. Preferred sweep rate was inversely proportional to the difference in synaptic delay of the outgoing connections from the tone-selective neurons (layer 1) to the output coincidence detection neurons (layer 2). This minimal model, using two layers of cells, can reliably encode rate and direction selectivity.

### Duration tuning

Duration tuned neurons are those that respond to a narrow range of tone durations, and are found across auditory regions and species. Duration tuning predicts FM sweep rate selectivity in both bats and mice [23], [24] on the basis that sweep rate will dictate how long a sound spends at each frequency in a sweep. This mechanism does not influence direction selectivity.

**Figure 6:**
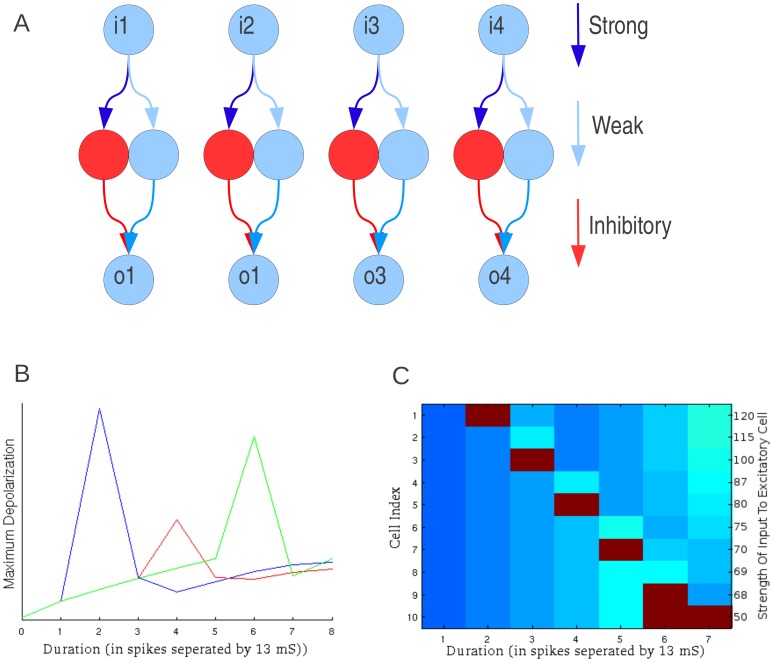
Duration tuning model mechanism of FM sweep detection. A) Cells of the output layer respond selectively to sweeps of a given duration of spike train fired by its respective input cell. Weak excitation is in pale blue; strong excitation is in dark blue. Inhibition is in red. Input to this network is in the form of spike trains that last the duration of the tone. The weight of the strong excitatory connection to the inhibitory cell is such that the inhibitory cell will fire roughly the same number of spikes over a similar duration as the input cell. The weak connection to the output cell is such that it will take a given number of input spikes before reaching threshold. The stage two excitatory cells connection to the output can be either sub threshold and require inhibitory rebound to reach threshold or supra-threshold. B) The maximum level of excitation achieved by output cells during a sweep as a function of input duration with spikes prevented to show maximum depolarization. C) This two dimensional plot shows maximum excitation of 10 output cells in response to a full range of input durations. Number of spikes is labeled across the x axis. Color indicates maximum excitation during stimulus presentation, While spikes are presented in deep red.

Published models of duration tuning depend on a tone onset driven early inhibition that lasts the duration of the tone and is followed by delayed excitation [25], [26]. The network implemented here uses these two components and consists of an input layer of tone-sensitive cells that fired a continuous train of spikes that lasted the input duration. Each input cell provides a strong excitatory input to a single middle layer inhibitory cell and a weak excitatory input to a middle layer excitatory cell. Inhibitory cells spiked with a similar spike rate and duration as the input cell and provided, with a delay, feed-forward inhibitory signal to the output cell. In contrast, the excitatory cell required many inputs to reach spike threshold and then provided excitatory input to the output cell (Fig. 6A). This results in an early onset-driven inhibition that lasts the duration of the tone and a delayed onset-driven excitation [25], [26]. When the input cell was activated for a duration within its preferred range, the output cell received excitatory input from the middle layer right after it stopped receiving inhibition. If this excitation exceeds threshold, spiking will occur. This will generate onset-dependent short-pass duration tuning functions for tones as predicted by the ‘anti-coincidence’ model of duration tuning [26]. In this model changing strength of the input to the middle layer excitatory cell would affect how long it takes to trigger spike in this cell and therefore will affect duration tuning of the circuit. Maximum excitation of the output cells from different circuits (with different strength of the input to excitatory neuron) is shown in Figs. 6B and 6C. In Fig. 6B the responses of 3 output cells are shown with spikes blocked. Each cell response showed a sharp peak for the preferred input duration (number of input spikes).

**Figure 7:**
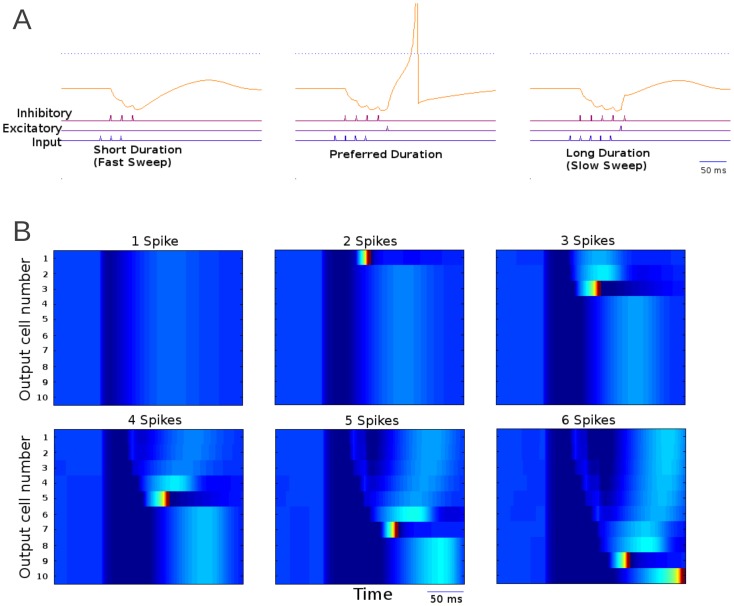
Duration tuned network response to a variety of sweeps. A) Cell traces of inputs one spike less than preferred duration (left), the preferred duration (mid) and one spike more than the preferred duration (right). The number of spikes reflects the tone duration. B) Raster plot of duration tuned output cells tuned to different durations (shortest preferred duration at top). The cells depolarization is represented by color. Cells are arranged along the y axis. Each frame represents the response to a single sweep of a different rate but similar bandwidth (thus a different duration of tone).

If the weak excitation does not reach threshold even if it is outside the window of early-onset inhibition, then a coincidence with a rebound from the inhibition may increase spiking probability (Fig. 7A, middle). This will lead to tone offset-dependent duration tuning as predicted by the ‘coincidence’ model of duration tuning [25]. When the input lasted too long, the inhibition was still present when the middle layer excitatory cell spiked. The inhibition cancelled this excitatory input and there was no response in the output cell (Fig. 7A, right). Finally, when the input duration was too short, the middle layer excitatory cell did not receive enough input to reach spike threshold (Fig. 7A, left). Thus the model explains two different hypotheses of tone duration tuning in the literature.

Fig. 7B shows the response of 10 output cells from different circuits (tuned to different durations) as a heat chart. Unlike the similar figures from the previous 2 models, here the inhibition triggers hyperpolarization that can be seen in dark blue starting significantly before the any of the output cells fire. Each neuron responded to a particular duration only. In this model, the sweep duration to which a given network is tuned was controlled by the strength of the excitatory connection from the input layer to the excitatory middle layer. The stronger this connection, the shorter the duration of the input triggering the network response. In natural environment, this would cause the network to respond to faster sweeps.

## Discussion

In this study we modeled mechanisms of FM sweep selectivity and identified network properties that may underlie the range of rate-dependent direction selectivity across species. In addition, we compared selectivity properties of these models and showed that STDP can shape selectivity in the sideband inhibition mechanism. The network models presented in our study are minimal sufficient networks to reproduce the main properties of FM mechanisms observed in vivo. The existence of all of these mechanisms in some species simultaneously indicates that each one likely possesses situational advantages over the others. Some of these advantages may be inferred from our study.

The facilitation mechanism is by far the simplest as it only requires 3 cells to create a circuit responsive to a single sweep rate and direction. It is, however dependent on the rate of synaptic decay to differentiate one sweep from another leading to trade off between specificity and response time that does not afflict the side band inhibition mechanism. There is also no obvious means to train this mechanism through synaptic plasticity as it depends upon differences in synaptic delay rather that synaptic strength. The sideband inhibition model is limited to fast pass selectivity unless additional layers of processing are available. The other two models do not contend with this issue. Sideband inhibition is also the most sophisticated of the mechanisms requiring the most frequencies of input, the most overall cells and precise timing of both excitation and inhibition. Primary advantage of duration selectivity mechanism over the others is its ability to estimate sweep rate without comparing the timing of multiple frequencies. It is however much slower than the other mechanisms and requires input in the form of spike trains.

### Sideband inhibition model

**Figure 8:**
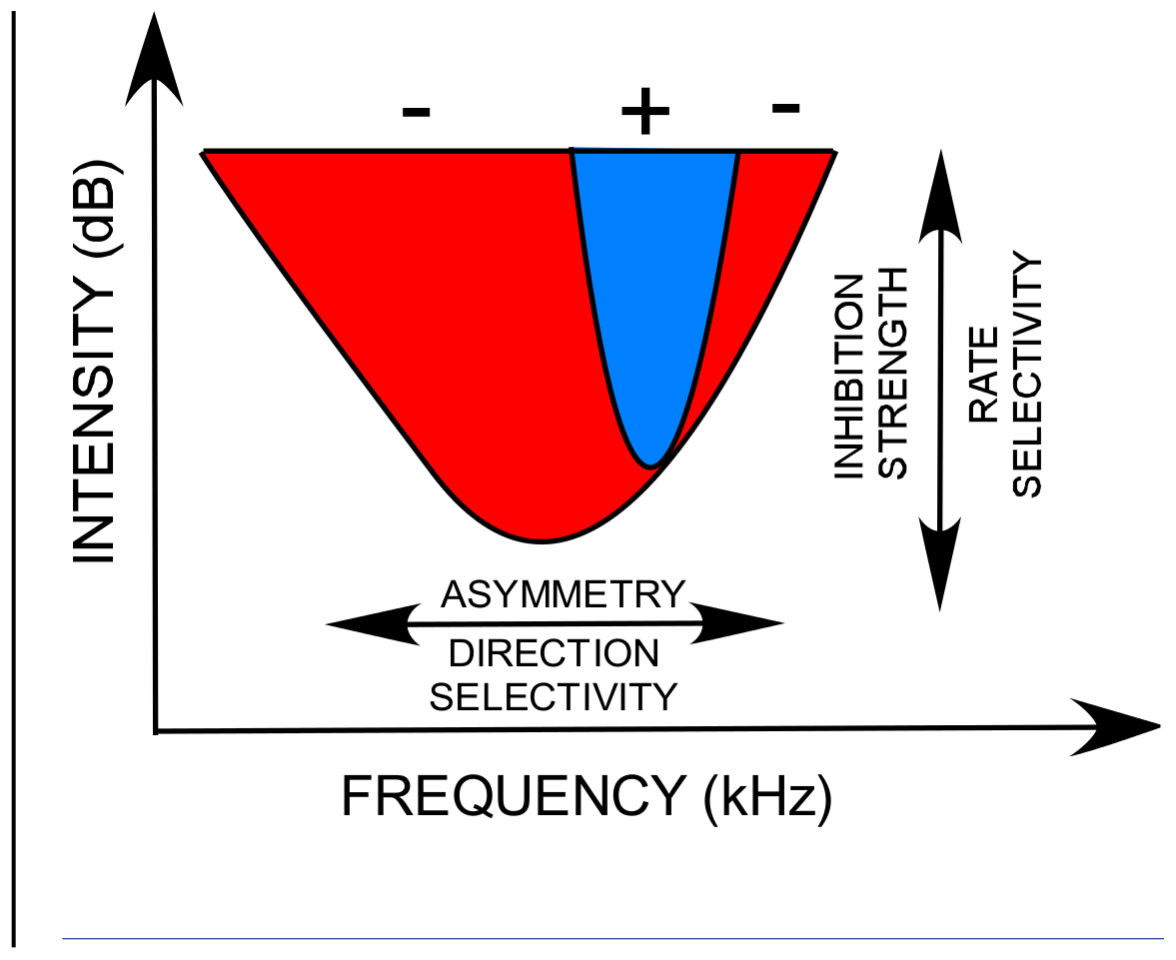
Schematic excitatory and inhibitory components of an auditory neuron frequency receptive field in a network with inhibitory sidebands. Variations in two properties, ‘asymmetry’ and ‘inhibition strength’, can explain the broad range of FM sweep rate-dependent direction selectivity observed across species. See text for additional details.

FM sweep rate and direction selectivity can be produced by the spectral and temporal interactions between excitation and sideband inhibition [9], [24], [35], [36], [37]. Our study shows that the diversity of rate-dependent direction selectivity functions observed across species can be explained by changes in two properties of the auditory neuron receptive field: *strength of excitatory input to inhibitory neurons that generate sidebands and the spectral asymmetry of these inputs* (Fig. 8). Variation along the abscissa generates differences in asymmetry and therefore generates differences in direction selectivity. Variation along the ordinate generates differences in relative strength of excitation and inhibition that may lead to the changes in relative timing of excitatory and inhibitory inputs to the output cell [17], [19] and, therefore, generates different ranges of sweep rates to which a neuron responds.

If the variation along the abscissa in Fig. 8 is related to tonotopy, this model explains the observed topography of FM direction selectivity in the rat auditory cortex in which low-frequency neurons preferred downward sweeps and high-frequency neurons preferred upward sweeps [20]. In the pallid bat cortex and inferior colliculus (IC), most neurons are selective for the downward FM sweeps used in echolocation [9], [38]. Analysis of timing of sideband inhibition in the cortex and IC showed that the high-frequency inhibition arrived later compared to low-frequency inhibition [9], [23]. This may also be due to the fact that the strength of high-frequency inhibition is lower compared to low-frequency inhibition as suggested by our model.

While we do not propose a complete model for the development of sideband inhibition based sweep detector, our study suggests that development of direction selectivity from non direction selective networks can be explained by STDP. Provided STDP has the largest impact on the connections from the input cells to the inhibitory cells, the network will prefer whatever sweeps it is exposed to most often. We found that to achieve successful training, STDP based plasticity required balancing of synaptic inputs such that the total synaptic strength per cell remained constant over the training. Total weight of synaptic inputs to a neuron could be conserved by local balancing of potentiation and depression [39] or by induction of heterosynaptic plasticity [40], [41], [42], [43], [44]. A similar model of direction selectivity in the ferret visual cortex has also come to the conclusion that synaptic competition is an essential component to obtain direction selectivity through synaptic plasticity [45].

Our model predictions are in agreement with experimental data obtained from the pallid bat cortex. At the onset of hearing most neurons are non-selective for sweep direction. During early development, the bat is likely exposed to echolocation calls within the colony. These are downward FM sweeps. Communication calls have upward sweeps, but are typically of slower rates or have different spectral bandwidths. The increased exposure to downward sweeps, through STDP, may increase the asymmetry (abscissa in Fig. 8) to favor responses to downward sweeps. Experimental data indeed show that during development low-frequency sideband inhibition arrives faster than high-frequency sideband [37] in an echolocation experience-dependent manner [12]. This is likely due to low-frequency sideband becoming stronger [19].

Several advantages of the sideband mechanism are apparent from the modeling results. This mechanism shows shorter response times than facilitation model. It was also less sensitive to small changes in synaptic strength when compared to facilitation mechanism. The most prominent disadvantage of the sideband mechanism is that it responds to a range of sweeps (fast-pass type selectivity functions). This can be corrected however by an additional layer of processing which subtracts the response of one network from another. An additional disadvantage to this mechanism is that it requires a broader range of frequencies in order to detect sweep rate. This creates problems with making the network capable of detecting properties of narrow bandwidth sweeps and in identifying sweep rate independent of sweep duration or starting frequency. Although the network described here can only produce a ‘fast pass’ filter that is capable of telling if a sweep is faster than a given rate, combining the outputs of several different networks tuned to slightly different sweep rates would allow creation of a ‘band pass’ network that only respond to a narrow range of sweep rates.

### Facilitation model

Facilitation (or coincidence detection) mechanism relies on differences in synaptic delay to derive sweep rate specificity. It is selective for both sweep rate and direction and responds only to a sweep within a narrow range of preferred rates. Only two inputs are required for any given detector cell and as such a tonotopic organization may not be necessary to form this detector. All that is required are cells that are responsive to a variety of tones. In a noiseless system the range of sweep rates that a given set of output cells would respond to can be made arbitrarily small.

This model has not been used previously to represent FM sweep processing, though models similar to this has been used to represent a sound localization system [46], [47] by detecting interaural time differences. The primary difference was that the inputs in the sound localization models represented the two ears rather than separate tones. As coincidence detection depended in part on decay of synaptic inputs to differentiate between sweeps, the time required to process a given input in this model was significantly longer (still <100 msec) than it was for the sideband inhibition model. Compared to the facilitation model, which could generate band-pass type (Gaussian) response functions, the sideband inhibition mechanism could only explain fast-pass (sigmoidal) FM rate selectivity without additional layers of processing.

It is very likely that a trade-off exists between selectivity for sweep rate and response time. As a network is made responsive to a wider range of sweeps by increasing the synaptic strength of the connection from the input cell, response times are likely to be reduced. The most likely cause of differences in synaptic delay is a difference in the placement of synapses upon the dendritic arbor; more distal synapses will take more time to carry signals on to the soma of the cell [48].

### Duration tuning

Two different models (coincidence and anti-coincidence based models) have been proposed to explain tone duration tuning. Common to both models is the presence of an early tone onset-driven IPSP and a delayed tone-onset driven EPSP. The early IPSP lasts the duration of the tone. The coincidence model posits that the EPSP is subthreshold and at the preferred duration, the rebound at the offset from inhibition will coincide with the EPSP to increase spiking. By definition, this mechanism will generate spikes that are tied to tone offset and such neurons are present in the IC. However, duration tuned neurons in which response is tied to tone onset are also present [23], [24], [26]. This can be explained by the anti-coincidence model that posits that the delayed EPSP is supra-threshold, and for short duration tones, the IPSP is over before the EPSP arrives. This will generate spikes. For long duration tones, the IPSP is still present when the EPSP arrives to reduce responses.

The network model implemented here explains both types of neurons based on the properties of a single connection from the excitatory input cell to another excitatory cell. Inhibitory rebound was not required for a duration-tuned network in this model. Nevertheless, well timed inhibitory rebound can cause some neurons to respond to what would otherwise be subthreshold inputs [49], [50]. Furthermore, inhibitory rebound may be able to increase signal to noise ratio. Recent work [51] developed a computational model of band pass duration tuned network. The model is based on the integrate-and-fire network and implemented populations of cells in six different nuclei. The mechanism of tuning was dependent upon either an offset evoked excitatory post-synaptic potential or inhibitory rebound. This finding differs from the model shown here, which can potentially function without either of these effects.

The most obvious disadvantage to this mechanism is that it is not inherently a sweep detector. It is, however, used as a sweep detector in the brain [23], [24]; in order to do this additional layers of processing are required. The key advantage of this mechanism is that it can identify a sweep using only a single tone as input. This makes it far more robust against certain types of damage, especially the loss of sensitivity to certain ranges of sound frequency as may occur with age-related hearing loss. This prediction is being tested in experimental models of cortical processing with hearing loss.

### Neural substrates of networks

The network arrangements of the mechanisms explored here are based on the stimulus-response properties of these mechanisms rather than attempting to replicate precisely any specific brain region. However, when guidance is available from published data, the circuits were designed to approximate biological reality. This is particularly true for the sideband inhibition model.

The structure of sideband inhibition network was derived from multiple studies of auditory cortical function. Local GABA-a receptor mediated inhibition shapes FM rate and direction selectivity in the auditory cortex justifying implementation of a cortex-based network with excitatory pyramidal cells and inhibitory interneurons [18]. The direct excitatory input from the input layer to the output layer mimics the thalamocortical excitatory input. The same inputs activate a feed-forward di-synaptic inhibition. Similar network structure was previously used in studies of thalamocortical processing [52], [53], [54]. In this network model the frequency selectivity and arrayed representation mimics tonotopy in both thalamus and cortex. The nature of connections result in both the inhibitory layer and the output layer to have similar spectral selectivity. This mimics the approximately balanced inhibitory and excitatory synaptic inputs that cortical neurons exhibit [55]. Finally, the asymmetries in spectral and/or temporal properties of sideband inhibition implemented here are also present in cortical neurons [9], [20].

The biological substrates of facilitation and duration tuning networks are less clear because few studies have explored the underlying synaptic circuits. Therefore these circuits were primarily based on response properties. Combination sensitive facilitation and duration tuning depend on local inhibitory activity in the IC. Application of antagonists of GABA-a and/or glycine receptors can reduce facilitation and duration tuning indicating that IC circuits are involved [26], [56]. Although we modeled facilitation as arising from two sub-threshold excitatory inputs, it is possible that at least one of these inputs is an inhibitory rebound excitation as has been shown in the IC [34]. It remains unknown if cortical circuits provide additional sharpening or *de novo* shaping of duration tuning and facilitation.

### Broader implications

FM sweeps are analogous to visual motion in the sense that the stimulus moves across the receptor epithelium. Mechanisms discovered in the auditory system, and implemented here, are similar to those present to extract visual motion direction and velocity information. For example, the cellular mechanism behind facilitation type FM sweep detector is similar to motion detection in the visual system [57]. The asymmetric sideband inhibition is similar to that present in the superior colliculus [58]. The duration tuning model is similar to that found in the visual cortex [59]. The developmental shaping of selectivity using STDP rules may also be similar in the two systems ([12], [28]). These models therefore are broadly applicable to examine synaptic mechanisms of sensory motion processing.

## Methods

All code used in these simulations is available at http://senselab.med.yale.edu/ModelDB/ShowModel.asp?model=168407.

### General definitions

The term ‘rate’ is used to describe the rate of frequency change in FM sweeps. Sideband inhibition refers to frequencies that by themselves do not produce action potentials, but can suppress spontaneous or excitatory tone-evoked responses. Facilitation refers to non-linear combination sensitive responses wherein, two tones in combination generate stronger responses than the sum of the individual tone responses. The main elements of the networks used for each model are outlined below and discussed in details in the Results section.

### Sideband inhibition model

The model is based on experimental data on sideband inhibition as a mechanism of FM rate and direction selectivity across species and brain regions [9], [14], [18], [19], [20], [23], [24], [35]. The spectral and temporal interactions between the excitatory and inhibitory components of the receptive field shape both direction and rate selectivity of the auditory neurons. Furthermore, any asymmetries of the inhibition on either side of the excitatory tuning curve will result in asymmetries in spectrotemporal properties and a rate-dependent preference for direction of stimuli moving across the frequency receptive field [9], [17], [19], [20], [35].

The excitatory component of the receptive field in the network model is a tonotopically-arranged layer of frequency-selective cells (Fig. 1A). Five adjacent cells in the input layer, each stimulated by a single frequency, stimulate a single cell in the output layer. The number of input cells represented was chosen arbitrarily and can be changed to represent sweep detectors with different tuning curve bandwidth. The same input cells connect to a cell in the inhibitory middle layer to simulate the approximately balanced inhibitory component of the receptive field [29], [30], [60]. The inhibitory middle layer cell strongly inhibits the output layer cell. Together these seven cells form the minimal unit of sweep detection with a distinctive profile of responses to sweeps in a single range of input frequencies. This profile of responses is dependent on the strength of the connections between the input cells and the inhibitory cell.

### Facilitation model

This model is based on experimental data on facilitation as a mechanism of FM sweep direction and rate selectivity across species and brain regions [21], [22], [34], [61]. Here the neuron's spiking response to individual tones in the receptive field is poor or absent. When, however, more than one synaptic input arrives simultaneously, it may lead to depolarization that is sufficient to initiate spiking response. This may occur when two tones are presented in an appropriately delayed sequence and activate two inputs with different synaptic delays. Direction selectivity for upward sweeps will emerge if an ascending, but not descending, tone sequence produces a necessary summation (also called facilitation). Rate-dependent response will emerge if only specific delays between tones produce facilitation.

In the implemented model, activity is stimulated in a tonotopically-arranged layer of frequency selective cells (Fig. 4A). Two cells in the input layer send subthreshold excitatory projections to a single output cell to model subthreshold narrowband excitatory inputs. The response from the excitation of both synapses combined is only sufficient to evoke a spiking response in the cell if they arrive within a narrow time window. Each synapse has a delay. In order for the excitation from both synapses to arrive within the time window the two input layer cells must fire with a timing that offsets the difference in delay between the synapses. In this model changing preferred sweep rate can be achieved by varying synaptic delay.

### Duration tuning model

This model is based on evidence for duration tuning as a mechanism of FM rate selectivity across species and brain regions [23], [24]. The different models for duration tuning have two elements in common: (1) a tone-onset driven early inhibition and (2) a delayed excitation [25], [26]. The models differ in whether coincidence of rebound from inhibition and excitation is necessary. We implemented a model to study the conditions under which rebound from inhibition may be necessary in shaping duration tuning. Activity is stimulated in a tonotopically-arranged layer of frequency selective cells. Each cell responds to an input with a continuous train of spikes for the duration of the input (Fig. 6A). An input layer cell sends a stronger excitatory connection to an inhibitory middle layer cell and a weaker excitatory connection to an excitatory middle layer cell. This will result in the early onset inhibition and delayed excitation. The inhibitory and excitatory middle layer cells send connections to the output cell. The output cell only responds to tones of specific duration. The preferred tone duration of this network is determined by the strength of excitation from the middle layer cell.
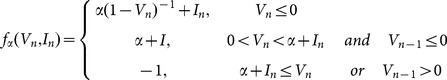



#### Individual neuron models

To allow for a detailed analysis of oscillatory dynamics in large-scale network simulations, we used a reduced neuron model described by difference equations (map) [62], [63], [64], which has a number of distinct numerical advantages: the common problem of selecting the right integration scheme is avoided since the model is already written in the form needed for computer simulations. The model is described by the following equations: 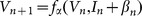 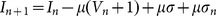, where *V_n_* is the membrane voltage, *I_n_* is a slow dynamical variable describing the effects of slow conductances, and *n* is a discrete time step (∼0.5 msec). Slow temporal evolution of *I_n_* was achieved by using small values of the parameter 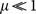. Input variables 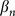 and 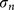 were used to incorporate external current 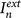 (e.g., synaptic input):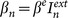, 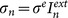. The nonlinearity 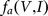 was designed in the form of a piece-wise continuous function. To convert the dimensionless “membrane potential” *V* to the physiological membrane potential *V_ph_*, the following equation was applied: *V_ph_* = V*50−15 [mV].

This model, despite its intrinsic low dimensionality, produces a rich repertoire of dynamics and is able to mimic the dynamics of Hodgkin-Huxley type neurons both at the single cell level and in the context of network dynamics(Rulkov, Timofeev et al. 2004; Bazhenov, Rulkov et al. 2005). Furthermore, these models are capable of matching response properties of biological neurons for more natural dynamic input [63] The network simulations conducted with this reduced model are several thousand times faster than those based on the Hodgkin-Huxley equations with a minimal set of ionic currents required to reproduce similar firing patterns of different cell types. In our simulations, the model parameters were set at 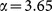, 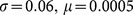, 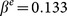, 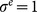. The model parameter 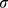 sets the resting potential of the neuron and, therefore, its state with respect to spiking threshold.

To model synaptic interconnections, we used conventional first order kinetic models of synaptic conductances rewritten in the form of difference equations:
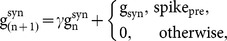
and the synaptic current computed as:
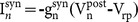
where *g_syn_* is the strength of synaptic coupling, and indices *pre* and *post* stand for the presynaptic and postsynaptic variables, respectively. The first condition, “spike*_pre_*”, is satisfied when presynaptic spikes are generated. Parameter 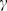 controls the relaxation rate of synaptic conductance after a presynaptic spike is received (0 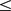 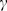 <1). Parameter *V_rp_* defines the reversal potential and, therefore, the type of synapse: excitatory or inhibitory. To model a synaptic delay, the implementation of condition “spike*_pre_*” may be delayed from the moment of the presynaptic spike generation by the number of iterations corresponding to the delay time. We used a range of 0.5–20 msec delays.

#### STDP modeling

STDP-based learning was implemented in the sideband inhibition model because this mechanism has received the most experimental support. Changes in sideband inhibition underlie developmental plasticity of FM sweep direction selectivity [12], [37]. When STDP-based learning was active, connections from the input layer to the middle inhibitory layer were plastic. To maintain balance of synaptic weights, the sum of all incoming connections to a given cell was held constant such that when one connection increases in strength all others correspondingly weaken. Plasticity in our model is based on a STDP paradigm [65]. A spike in a post-synaptic cell which directly follows a spike in pre-synaptic cell creates a “*pre before post*” event. Additional post-synaptic spikes do not create additional *pre before post* STDP events. Likewise a spike in a pre-synaptic cell which directly follows a spike in post-synaptic cell creates a “*post before pre*” event. Additional pre-synaptic spikes do not create additional *post before pre* STDP events.

The value of an STDP event was calculated using the following equation [66], [67]:
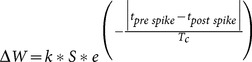
Where *k* is equal to −0.025 in the case of a *post before pre* event and 0.025 in the case of a *pre before post* event. Variable *S* is the strength of the connection. *T_c_* is the time constant and is equal to 10 ms.
