# Correction: Network Models of Frequency Modulated Sweep Detection

**DOI:** 10.1371/journal.pone.0136010

**Published:** 2015-08-14

**Authors:** Steven Skorheim, Khaleel Razak, Maxim Bazhenov

The following information is missing from the Funding section: This study was supported by Office of Naval Research Multidisciplinary University Research Initiative Grant N000141310672. The funders had no role in study design, data collection and analysis, decision to publish, or preparation of the manuscript.
